# The effects of inhaled aztreonam on the cystic fibrosis lung microbiome

**DOI:** 10.1186/s40168-017-0265-7

**Published:** 2017-05-05

**Authors:** Alya A. Heirali, Matthew L. Workentine, Nicole Acosta, Ali Poonja, Douglas G. Storey, Ranjani Somayaji, Harvey R. Rabin, Fiona J. Whelan, Michael G. Surette, Michael D. Parkins

**Affiliations:** 1 Department of Microbiology, Immunology and Infectious Diseases, The University of Calgary, Calgary, AB Canada; 2 Faculty of Veterinary Medicine, The University of Calgary, Calgary, AB Canada; 3 Department of Biological Sciences, The University of Calgary, Calgary, AB Canada; 4 Department of Medicine, The University of Calgary, 3330 Hospital Drive, NW, Calgary, AB Canada; 5 Department of Biochemistry and Biomedical Sciences, McMaster University, Hamilton, ON Canada

**Keywords:** Microbiota, AZLI, Inhaled antibiotics, *Pseudomonas aeruginosa*, *Prevotella*, *Fusobacterium*

## Abstract

**Background:**

Aztreonam lysine for inhalation (AZLI) is an inhaled antibiotic used to treat chronic *Pseudomonas aeruginosa* infection in CF. AZLI improves lung function and quality of life, and reduces exacerbations-improvements attributed to its antipseudomonal activity. Given the extremely high aztreonam concentrations achieved in the lower airways by nebulization, we speculate this may extend its spectrum of activity to other organisms. As such, we sought to determine if AZLI affects the CF lung microbiome and whether community constituents can be used to predict treatment responsiveness.

**Methods:**

Patients were included if they had chronic *P. aeruginosa* infection and repeated sputum samples collected before and after AZLI. Sputum DNA was extracted, and the V3-hypervariable region of the 16S ribosomal RNA (rRNA) gene amplified and sequenced.

**Results:**

Twenty-four patients naïve to AZLI contributed 162 samples. The cohort had a median age of 37.1 years, and a  median FEV_1_ of 44% predicted. Fourteen patients were *a priori *defined as responders for achieving ≥3% FEV_1_ improvement following initiation. No significant changes in alpha diversity were noted following AZLI. Furthermore, beta diversity demonstrated clustering with respect to patients, but had no association with AZLI use. However, we did observe a decline in the relative abundance of several individual operational taxonomic units (OTUs) following AZLI initiation suggesting that specific sub-populations of organisms may be impacted. Patients with higher abundance of *Staphylococcus* and anaerobic organisms including *Prevotella* and *Fusobacterium* were less likely to respond to therapy.

**Conclusions:**

Results from our study suggest potential alternate/additional mechanisms by which AZLI functions. Moreover, our study suggests that the CF microbiota may be used as a biomarker to predict patient responsiveness to therapy suggesting the microbiome may be harnessed for the personalization of therapies.

**Electronic supplementary material:**

The online version of this article (doi:10.1186/s40168-017-0265-7) contains supplementary material, which is available to authorized users.

## Background

Cystic fibrosis (CF) is characterized by chronic lung infections and persistent inflammation resulting in progressive airways disease. Although CF is typified by a number of classical bacterial infections, *Pseudomonas aeruginosa* represents the archetypal CF pathogen. Ultimately, 60–80% of CF patients will become chronically infected with *P. aeruginosa* [1]. Chronic infection with *P. aeruginosa* and its conversion to an alginate hyper-producing mucoid phenotype is associated with progressive decline in lung function, worsening quality of life, increased risk of hospitalization, and reduced survival [2]. Accordingly, novel management strategies for both acute and chronic infections have been at the forefront of efforts to improve outcomes in CF. The concept of aerosolized antibiotics as a means of delivering potent therapy to the lower airways was first proposed in 1946; however, it has only been widely implemented in the last three decades [3]. Aerosolized delivery of antipseudomonal antibiotics allows for the deposition of supra-therapeutic levels of drugs directly to infected airways. This may overcome many intrinsic and adaptive resistance mechanisms of *P. aeruginosa* while avoiding systemic exposure to high levels of the medication and its resultant toxicity [4].

One such agent, aztreonam lysine for inhalation solution (AZLI) (Cayston®, Gilead Sciences), has been licensed for use in CF patients with chronic *P. aeruginosa* lung infection in a cyclical fashion of 28 days on/off drug [5–9]. Studies have demonstrated that AZLI improves clinical outcomes in CF including lung function, quality of life indices, weight gain, and delayed time to next pulmonary exacerbation [5–9]. In a 6-month comparative trial, AZLI exhibited superior effects compared to tobramycin inhalation solution (TIS) in improving lung function and reducing exacerbation frequency [6]. Improvements in CF patients prescribed inhaled antibiotics, and AZLI in particular, have long been presumed due to their antipseudomonal cidal effects and resultant reduction in *P. aeruginosa* sputum density (approximately 1.5 log10 colony-forming units (CFU)/g of sputum during cycles on therapy) [5–9]. However, *P. aeruginosa* persists at a range of 5.5–6.0 log10 CFU/g of sputum despite potent inhaled antibacterial therapies. Furthermore, no correlation has ever been demonstrated between improvements in lung function and decline in *P. aeruginosa* density [3, 5–9]. This suggests that these inhaled antibiotics may have additional unknown mechanisms of action.

Recently, a complex polymicrobial community of microorganisms residing in the lower airways referred to as the CF lung microbiota has been recognized [10]. Many of these organisms are specifically not cultured or ignored entirely through traditional culture-based approaches [11]. However, the role of the CF microbiome at this point in health and disease is relatively unknown. Unfortunately, studies regarding the microbiome have generally been limited to short-term longitudinal or cross-sectional cohort studies [12, 13]. Importantly, components of the microbiome (such as the *Streptococcus anginosus* group) have been associated with disease pathogenesis [14], while other organisms are thought to protect the microbiota by the production of antibiotic degrading enzymes [15, 16]. The administration of systemic antibiotics has been shown to have dramatic, albeit transient, effects on the microbial community composition [17]. However, information as to whether the microbiome influences outcomes or therapeutic response remains limited.

Commonly used inhaled antibiotics include aztroenam, tobramycin and colistin, and are traditionally considered to impact only aerobic Gram-negative organisms, based on drug levels achieved during systemic administration. However, we speculate that the exceedingly high concentrations achieved through aerosolization may extend their spectrum of activity to other members of the CF microbiota including Gram positives and anaerobes [18]. Indeed, it may be that several existing CF therapies exert their therapeutic response through the manipulation of the microbiome. We hypothesized that at least a portion of AZLI’s beneficial effect is microbiome mediated. To determine if this may be the case, we sought to define the changes induced in the CF microbiome following the initiation of AZLI and correlate whether microbiome composition influenced response to therapy.

## Methods

### Patients and sample collection

Since 1997, the Calgary Adult Cystic Fibrosis Clinic (CACFC) has kept and maintained a frozen repository of sputum from adult CF patients submitted for clinical assessment. This biobank includes >13,300 sputum samples from 275 adult CF patients collected as part of their ongoing clinical care. All patients provide prospective consent for the collection and storage of sputum samples for research purposes as approved by the Calgary Health Region Ethics Board (REB15-0854). Patients were included in the study if they had chronic *P. aeruginosa* infection as defined by the Leeds criteria [19], received AZLI for at least 1 year, and had at least two sputum samples within 1 year before (pre-samples) and 1 year after the initiation of AZLI (post-samples) available for analysis in the CACFC biobank. Day 0 samples were included in the pre-AZLI category.

Patient demographics assessed included age, gender, age at diagnosis, pancreatic status, and genetics. Dynamic variables of disease at the time of collection were recorded including lung function (percent predicted forced expiratory volume in 1 s (FEV_1_) and percent predicted forced vital capacity (FVC)) as determined using the Knudson calculation, nutritional status (body mass index (BMI) (kg/m^2^)), cultured classical CF pathogens, and chronic therapies. Clinical notes were reviewed to identify any documentation indicating if patients were currently administering the inhaled antibiotic and which day of the cycle they were on.

Concurrent therapies at treatment initiation and through the course of AZLI, including inhaled antibiotics and acute therapies for pulmonary exacerbations, were recorded. Pulmonary exacerbations were clinically defined events; however, previous work from our group has demonstrated 100% concordance with Fuchs’ criteria [20, 21]. To determine changes in lung function following the initiation of AZLI, FEV_1_% values recorded in the year before and after AZLI measured during periods of clinical stability were modeled using a linear mixed effects model. Periods of clinical stability were defined as samples free of systemic therapies for >1 week and free of pulmonary exacerbation. We have purposefully selected a 1-week time frame as systemic antibiotics have been shown to have transient effects on the microbiota and community composition returns to baseline within approximately 1 week [12, 13, 17, 22]. Patients were differentiated into AZLI “responders” and “non-responders.” The definition of responder was a priori defined as achieving an improvement in FEV_1_ of ≥3% predicted over the year (excluding values obtained during pulmonary exacerbations) following AZLI initiation, based on evaluation of clinical study data where short-term improvements of 2.7–10.8% were observed [5, 6, 8, 9].

### DNA extractions & 16S rRNA amplification and processing

Microbial community profiling of sputum samples was conducted as previously described [23]. Amplification of the V3 hypervariable region of the 16S ribosomal RNA (rRNA) gene was carried out using reverse and forward barcoded primers using the Illumina MiSeq technology at the McMaster Genome Facility (Hamilton, ON). Reagent blanks are run for each set of DNA extractions, and samples are excluded if these controls are positive for PCR products and DNA extractions repeated as necessary. Sequencing reads were analyzed using custom Perl scripts [24]. Briefly, sequences with low quality reads were trimmed using Cutadapt [25]. PANDAseq was used to assemble paired-end reads [26]. Operational taxonomic units (OTUs) were picked using AbundantOTU+ for OTUs with ≥97% identity [27]. The RDP classifier (version 2.2) was used to assign taxonomy against the Greengenes reference database [28, 29]. Analysis was carried out using QIIME (version 1.8.0) and Phyloseq (version 1.16.2) R package [30, 31].

### Microbial community composition and statistical analysis

Microbial communities were assessed based on the below discrete categorical variables. Samples collected prior to treatment (pre) were compared with those collected after treatment (post) to determine how AZLI impacts the microbiome of a naïve patient population. Post-samples were further stratified based on whether they were collected during “on” vs “off” cycled AZLI therapy. Those samples collected within 7 days of parenteral antibiotics for a pulmonary exacerbation were assessed for changes associated with systemic therapies and excluded from other analyses to limit confounding effects [17]. In order to determine if there are biomarkers associated with clinical response to AZLI, the microbiome of responders vs non-responders was also compared. Finally, we analyzed whether there were gender-based differences in the microbiome as an attempt to better characterize the gender gap in CF lung disease [32]. Wilcoxon rank-sum tests and paired *t* tests were used to test for significant differences in alpha diversity measures of observed OTUs and Shannon diversity indices (SDI), in the phyloseq package in R [31, 33]. Community structures were assessed using Bray-Curtis (BC) beta diversity measures after proportionally normalizing all samples as previously described [12]. Permutational multivariate analysis of variance (PERMANOVA) was used to analyze statistical differences in beta diversity using the vegan package in R [33]. Results were visualized using principal coordinate analysis (PCoA) plots. In addition to community-wide differences, we were also interested in differences at the genus and OTU level; Wilcoxon rank-sum tests and paired *t* test were employed to calculate differences in the relative abundance values of organisms present in >20% of samples for the different categorical variables being assessed. Log abundance plots were used to visualize these results using ggplot2 package in R [34]. Samples with an abundance value of 0 were changed to a value of 1 since log(0) is undefined and log(1) = 0. As a sensitivity analysis, a linear mixed model with random intercepts was constructed to examine differences in diversity as well as relative abundance of organisms present in >20% of samples (similar to above) for the pre- and post-AZLI period. Secondly, a restricted analysis (using only one sample/patient/category) of the observations closest to initiation of AZLI in the pre- and post-periods was conducted utilizing paired *t* tests and Wilcoxon rank-sum tests for the previously detailed tests as a comparator.

## Results

### Patient and sample characteristics

Twenty-four patients met the defined study inclusion criteria. Patient characteristics, concurrent therapies, and conventionally cultured pathogens at initiation of AZLI (baseline) are listed in Tables 1 and 2. The cohort was generally composed of individuals with more advanced disease given AZLI’s role as a salvage agent within Canada [35]. Median lung function as measured by FEV_1_% predicted at initiation of AZLI for the cohort was 44.0% (IQR 33.0–52.75). Prior to the initiation of AZLI, 21/24 (87%) patients were maintained on inhaled tobramycin and 4/24 (16%) inhaled colistin. Following initiation of AZLI, 17/24 (71%) patients continued on AZLI as their sole inhaled antipseudomonal antibiotic (in 28-day on/off cycles) and 7/24 (29%) continued on chronic sequential aerosolized antibacterial therapies alternating with TIS (tobramycin inhalation solution) (3/24), TIP (tobramycin inhalation powder) (2/24) or colistin (2/24) (Additional file 1: Figure S1).

**Table 1:** Patient characteristics for cohort (*n* =24) being studied. Values were taken at the initiation of AZLI

Characteristics of patient cohort	Responders (*n* = 14)	IQR	Non-responders (*n* = 10)	IQR	*p* value
Female sex	9		3		0.24
Median age, years	41.0	35.0–46.0	40.0	32.0–44.0	0.4
Median age at diagnosis, years	2.8	1.9–11.5	0.3	0.1–0.5	0.04
BMI (Kg/m^2^)	*21.6*	20.9–22.7	21.9	18.9–24.8	0.8
Lung disease status					
Mild FEV_1_ (≥70%)	1		1		
Moderate FEV_1_ (40–70%)	7		3		
Severe FEV_1_ (≤40%)	6		6		
^a^Median FEV_1_ (%) predicted at initiation	46.0	*33.0*–*57.0*	38.5	33.0–50.0	0.4
^a^Median FEV_1_ (%) predicted post-initiation	45.0	35.0–57.0	33.5	30.0–48.0	0.2
Genotype					
ΔF508 homozyogous	8		6		
ΔF508 heterozygous	3		3		
CF related co-morbidities					
Pancreatic insufficiency	12		10		
CF-related diabetes	4		4		
Impaired glucose tolerance	5		3		
Liver disease	1		1		
Bone disease	5		3		
Sinus disease	6		3		
Recurrent distal intestinal obstructive syndrome (DIOS)	4		1		
Cultured Pathogens					
*P. aeruginosa*	14		10		
*E. coli*	1		0		
Methicillin susceptible *S. aureus*	0		3		
Group B streptococcus Spp*.*	1		0		

^a^Represents the median of all spirometry values recorded in the year including those where a pulmonary exacerbation occurred

**Table 2:** Concurrent therapies taken by patient cohort at the initiation of AZLI as part of their routine care

Medication	Responders (*n* = 14)	Non-responders (*n* = 10)
Nutritional		
CF specific multi-vitamin	14	10
Vitamin D	14	10
Pancreatic enzymes	12	10
Chronic oral antibiotic therapies		
Azithromycin	11	9
Ciprofloxacin	2	2
Other antibiotics^a^	0	3
Inhaled antibacterial cycling		
AZLI sole inhaled antibiotic	12	5
Tobramycin supplemented (TIS/TIP)	1	4
Colistin supplemented (colomycin)	1	1
Respiratory		
Short-acting beta-agonist	11	9
Long-acting beta-agonist	14	9
Inhaled corticosteroid	8	2
Nasal steroid	5	1
Dornase alpha (DNase)	10	8
Hypertonic saline	8	6
Gastrointestinal		
PPI (pantoprazole/omeprazole)	4	6
Ursodiol	2	1
Ultratums	12	10
Laxative	3	4
Endocrine		
Oral birth control pill	2	1
Insulin	4	4
Oral hypoglycemic	1	2
Other		
Antidepressant	5	3
NSAID	0	1

^a^Other antibiotics include amoxicillin, cefotaxime, and septra

**Fig. 1:**
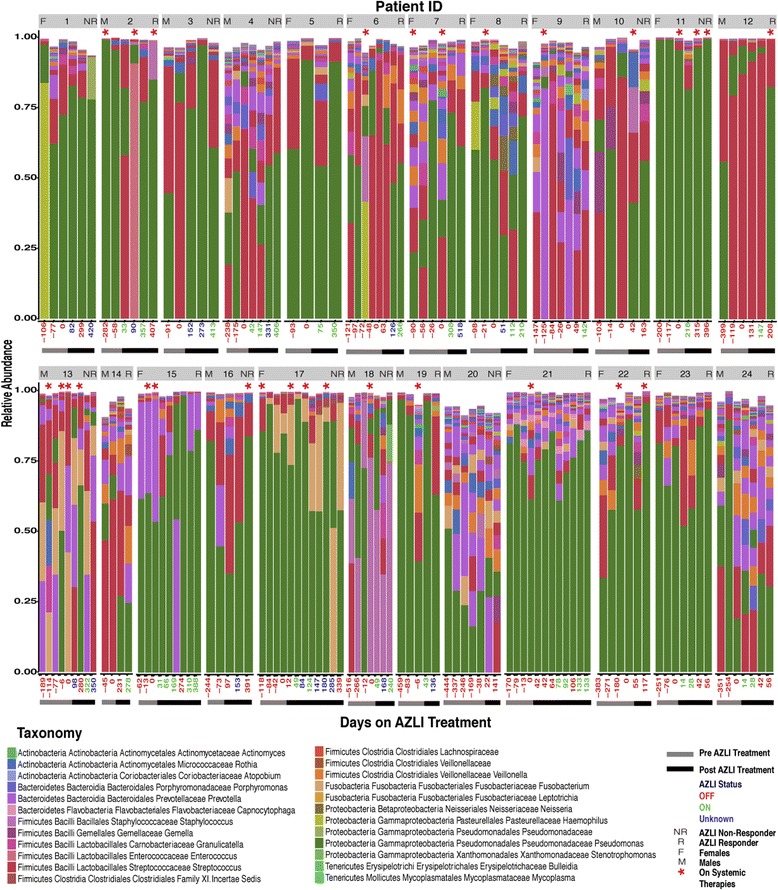
Taxonomic summaries of taxa present in >1% of samples (*n* = 162) for a cohort of 24 CF patients with chronic *P. aeruginosa* infection as a function of days from the initiation of AZLI treatment. *Grey* and *black bars* below plots represent samples pre- and post- the initiation of AZLI, respectively. Days from the initiation of AZLI are represented on the *x*-axis; *red text* = off AZLI, *green* = on AZLI, *blue* = unknown AZLI status. Patient ID, gender, and response status are listed on each bar plot. Samples on systemic antibiotics (*asterisks*). Analysis of the microbiome reveals unique profiles associated with each patient and varying abundance of *P. aeruginosa* over time

One hundred and sixty-two sputum samples were included (median 6/patient (IQR 5–7)) representing before (*n* = 80) and after (*n* = 82) the initiation of AZLI (Fig. 1). Of the samples collected following the initiation of AZLI, 35/82 were collected during a 28-day on cycle, 29/82 collected during an off cycle, and 18/82 were unknown as day of cycle was not recorded in the clinical records (Fig. 1). Samples *n* = 29 (17 pre- and 12 post-) collected during the treatment of a pulmonary exacerbation were excluded from some analyses owing to confounding effects of systemic antibiotics.

### Significant heterogeneity in the microbial community composition of the lower airways was observed amongst the cohort

A total of 15,029,112 sequences (median per sample 80,242 (IQR 27,432–134,552)) were generated from the 162 sputum samples assessed. Taxonomic summaries were used to visualize changes in the microbiota before and after the initiation of AZLI for taxa present in >1% of all samples (Fig. 1). Patient ID, gender, response status, and sample details including pre- vs post-AZLI, on vs off AZLI, and on systemic therapies are included in Fig. 1. These community-wide profiles display marked inter-patient heterogeneity. For example, although *Pseudomonas* was identified in all samples, the relative abundance was variable, with a median abundance of 56% (IQR 17–80%). Moreover, while some patients (for example, 11 and 21) were dominated by *Pseudomonas*, others (for example, 9 and 13) had a more diverse microbiome composed of organisms belonging to several different genera.

### Changes induced in the microbiome following initiation of AZLI

**Fig. 2:**
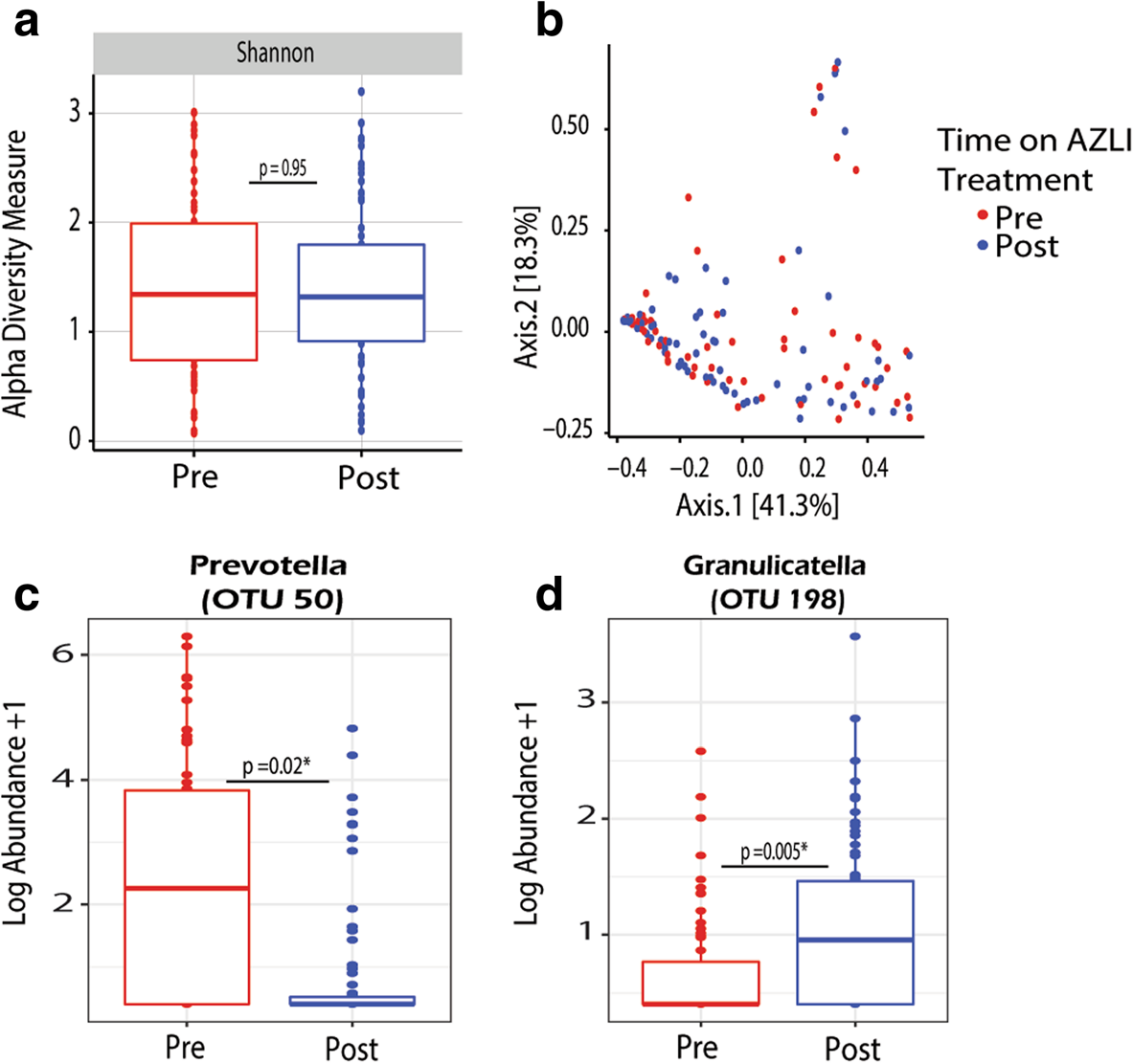
Shifts in the microbiome of samples collected prior (*n* = 62) and post- (*n* = 70) initiation of AZLI, samples on systemic therapies were filtered out. **a** Differences in Shannon diversity index prior to and post-initiation of AZLI as measured by a paired *t* test. **b** Bray-Curtis-based PCoA plot showing community-wide differences of samples collected prior (*red*) and post- (*blue*) initiation of AZLI. PERMANOVA reveals no community-wide differences between the samples collected prior to and post-initiation of AZLI (*p* = 0.52). **c**–**d** Paired *t* test revealed significant differences in the log abundance of individual OTUs in the samples collected prior vs post-initiation of AZLI, for organisms present in greater than 20% of samples being assessed

From our samples, no differences were noted in alpha diversity in those samples collected prior to and post-initiation of AZLI, even when samples were filtered on the basis of receipt of systemic therapy (Fig. 2a). A linear mixed model resulted in similar diversity values in the pre- and post-periods. Furthermore, no significant difference in beta diversity between pre- and post-AZLI samples was observed in Bray-Curtis PCoA plots and PERMANOVA when analyzed in aggregate (*p* = 0.52) or when conducted by setting constrains to the permutations by patient to control for potential confounders (*p* = 0.68) (Figure 2B). However, in samples collected post-AZLI initiation, there was a significantly lower relative abundance in *Prevotella* and higher relative abundance of *Granulicatella*, although the latter was present in low levels (Figure 2c, d). Sensitivity analysis using restricted numbers of observations demonstrated similar findings although *Prevotella* is no longer significantly different. The linear mixed models provided similar results with regards to the SDI, *Prevotella*, and *Granulicatella* in the pre- and post-periods.

**Fig. 3:**
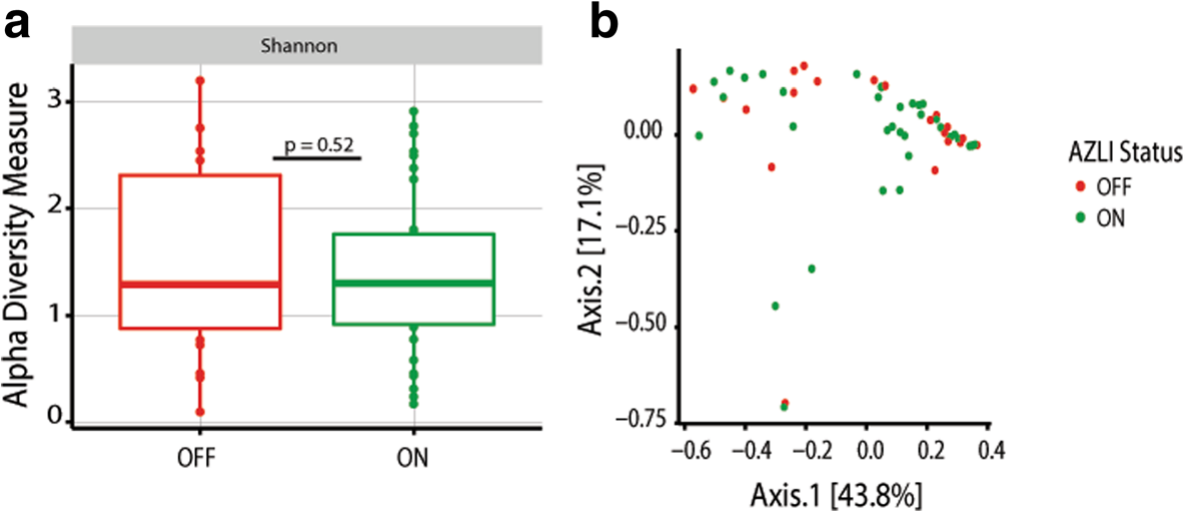
**a** Analysis of samples collected post- (*n* = 55) the initiation of AZLI reveal no significant differences in **a** Shannon alpha diversity measures as determined using a paired *t* test. **b** Bray-Curtis beta diversity measures between samples collected ON (*n* = 35) and OFF (*n* = 20) cycled AZLI therapy show no significant differences as demonstrated by PERMANOVA (*p* = 0.89). Comparably, when permutations are constrained by patient the *p* value is quite similar (*p* = 0.88). Similarly, no individual OTUs were found to be significantly different. Samples on systemic therapies were filtered out of the assessment

Furthermore, when we assessed changes in the microbial communities of samples post-initiation of therapy based on whether they were collected during on (*n* = 35) vs off (*n* = 29) cycle of AZLI, differences were not observed in SDI and samples did not cluster in Bray-Curtis PCoA plots when analyzed in aggregate (*p* = 0.89) (Fig. 3a, b) or when restricted to those patients who had matched samples (*p* = 0.88). Moreover, no significant changes were observed when analyzing organisms at the genus and OTU levels.

### Changes in the microbiome associated with clinical response to AZLI

**Fig. 4:**
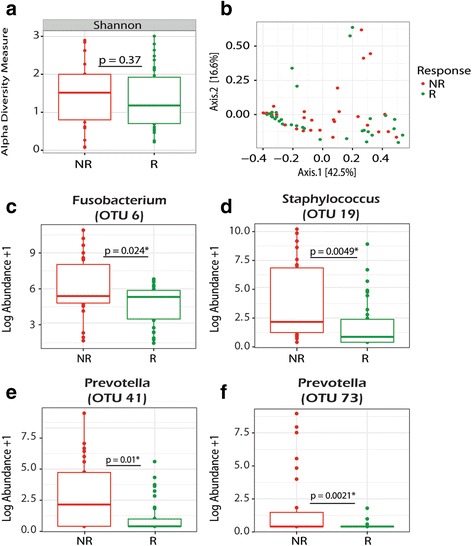
Analysis of shifts in the microbiome of samples collected prior to the initiation of AZLI (excluding those on systemic antibiotics) taken from responders (R) (*n* = 35) vs non-responders (NR) (*n* = 28). **a** Shannon alpha diversity measures comparing samples from R and NR. **b** PCoA plot showing community-wide differences of samples taken from R vs NR as measured by Bray-Curtis beta diversity measures. PERMANOVA reveals no significant community-wide differences in aggregate (*p* = 0.49) or when permutations were constrained by patient (*p* = 0.85). **c**–**f** *Box plots* showing significant differences in the log abundance of organisms at the OTU level present in >20% of samples taken from R vs NR. Wilcoxon rank-sum tests reveal significant differences in organisms belonging to four distinct OTUs

**Fig. 5:**
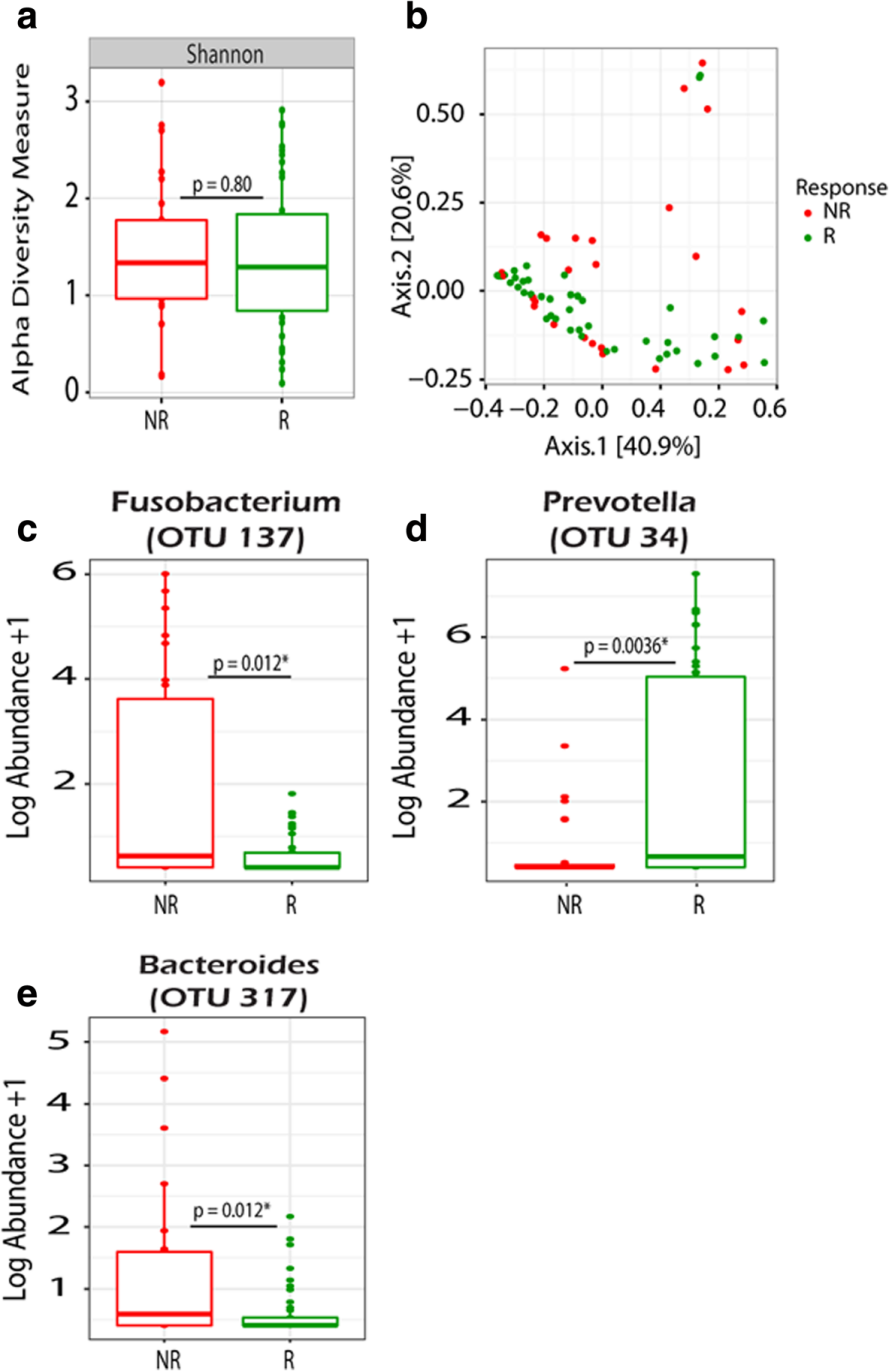
Assessment of microbiome differences POST AZLI initiation in responders (R) (*n* = 54) vs non-responders (NR) (*n* = 16). **a** No significant differences were observed in Shannon diversity index. **b** Similarly, no significant differences were in Bray-Curtis PCoA plot as measured by PERMANOVA (*p* = 0.06). When permutations were constrained by patient, the *p* value increased dramatically to (*p* = 1.0). **c**–**e** *Box plots* showing significant differences, as demonstrated by the Wilcoxon rank-sum test, in the log abundance of organisms identified at the OTU level present in >20% of samples. Samples on systemic therapies were filtered out

We further sought to determine if the microbiome may be used as a biomarker to identify patients more or less likely to demonstrate a clinical improvement on AZLI. According to our *a priori* definition, 14/24 patients achieved a ≥3% improvement in lung function following the initiation of AZLI. Number of exacerbation events in the year following AZLI did not differ between responders and non-responders (median 0 vs 1, *p* = 0.66) nor did total days of IV antibiotics (median 9 [IQR 0–26] vs 16 [IQR 0–32], *p* = 0.6). When comparing only pre-samples (excluding those on systemic therapies), we found no significant shifts in SDI or Bray-Curtis beta diversity measures using PERMANOVA (*p* = 0.49) (Fig. 4a, b). However, when response was stratified by female gender, there was significance (*p* = 0.02) suggesting gender is a confounder. When permutations were constrained by patient, the *p* value increased (*p* = 0.85) (Fig. 4b). Next, we sought to identify changes at the genus and OTU level. Non-responders trended towards lower abundance of *Pseudomonas* 43.0% (IQR 12.9–72.8%) compared to responders 57.0% (14.4–81.4%) (*p* = 0.61). Non-responders had a higher abundance of *Staphylococcus, Fusobacterium*, and *Prevotella* (Fig. 4c–f). When restricted analyses were conducted, *Fusobacterium and Prevotella* (OTU 41) were no longer significantly different. No significant differences were detected in SDI (Fig. 5a) or Bray-Curtis beta diversity measures using PERMANOVA (*p* = 0.07) (Fig. 5b) in samples collected after AZLI initiation. Furthermore, when permutations were constrained by patient, the *p* value increased dramatically to *p* = 1.0 suggesting that differences may be derived by patient rather than response status (Fig. 5b). Higher abundances of *Fusobacterium* and *Bacteroides* following treatment initiation were also associated with lack of clinical response (Fig. 5c, e). Responders had a higher abundance of *Prevotella*, although it was not the same OTU (34 rather than 41 and 73) associated with lack of response in the pre-sample assessment (Figs. 4e, f and 5d). Restricted analyses demonstrated similarly that SDI was comparable following AZLI initiation in responders and non-responders.

### Patient demographics associated with changes in the microbiome

**Fig. 6:**
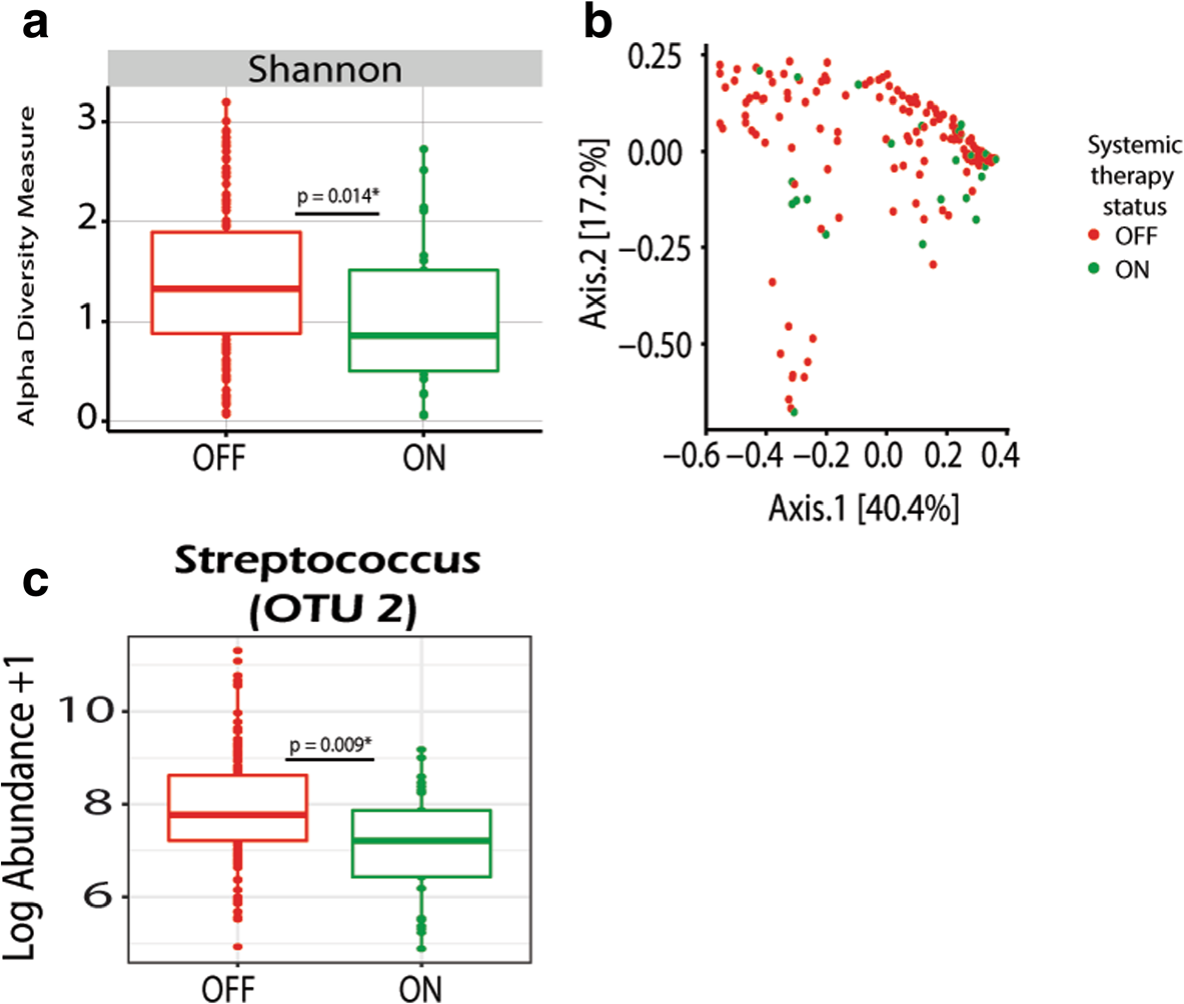
Analysis of samples collected ON (*n* = 29) vs OFF (*n* = 132) systemic antibacterial therapies. **a** Significant differences were found in Shannon alpha diversity measures as measured by a paired *t* test. **b** Bray-Curtis PCoA plot of samples ON vs OFF systemic therapies. PERMANOVA reveals significant community-wide differences (*p* = 0.02*), as shown in the PCoA plot. Even when permutations are constrained by patient significance is observed (*p* = 0.04). **c** Significant differences in the log abundance of samples ON vs OFF systemic therapies were detected in *Streptococcus* (OTU2) using a paired *t* test

**Fig. 7:**
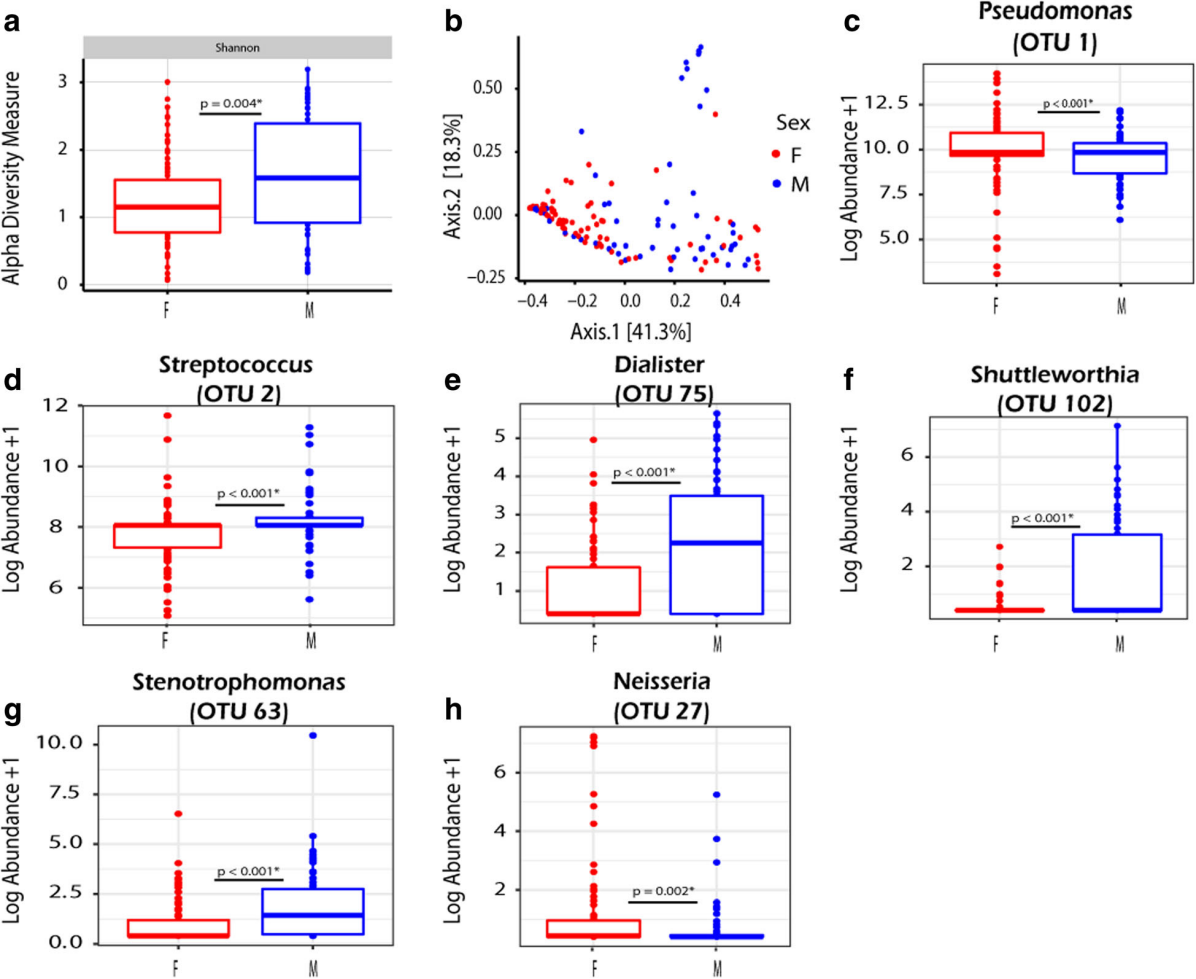
Analysis of samples collected from males (*n* = 56) vs females (*n* = 76). **a** Males have a higher alpha diversity measure as measured by the Wilcoxon rank-sum test. **b** Similarly significant differences were observed in PERMANOVA testing (*p* = 0.001*) as demonstrated by clustering in the Bray-Curtis PCoA plot. However, significance is lost when permutations are constrained by patients (*p* = 1.0). **c**–**h** Significant differences in the log abundance at the OTU level for organisms present in >20% of samples, as demonstrated by Wilcoxon rank-sum statistical analyses

We assessed the association of gender and systemic therapies on the microbiome. We identified that both SDI and community structure of samples on vs off systemic antibiotics were significantly different (Fig. 6a, b). This was true even when permutations were constrained by patient (*p* = 0.04). Furthermore, samples collected during use of systemic therapies had significantly reduced levels of *Streptococcus*. Gender analysis revealed that samples collected from males had a significantly higher SDI; this correlated with a reduced proportional abundance of *Pseudomonas* and increased abundance in other genera including *Streptococcus, Dialister*, *Shuttleworthia*, and *Stenotrophomonas* (Fig. 7). Males also had a higher abundance of *Rhodanobacter* and *Tepidimonas*; however, these organisms were present in very low abundance. Conversely, females had a higher abundance of *Pseudomonas* and trended towards improved responsiveness to AZLI: 9/12 vs 5/12 (*p* = 0.24). Community-wide differences as measured by PERMANOVA show significant differences (*p* = 0.001) between males and females (Fig. 7b). However, when constraints to permutations are set by patient in the PERMANOVA, the *p* value becomes 1.0 suggesting these differences may be due to inter-patient variability in the microbiome (Fig. 7b). Sensitivity analyses using restricted observations were similar with the primary results; however, SDI and the abundance of *Stenotrophomonas and Neisseria* were no longer significantly different.

## Discussion

In the last decade, scientists have come to recognize that CF airways are infected by a polymicrobial community of organisms beyond those classical CF pathogens [10, 36]. However, the role of these complex communities remains to be elucidated. While the CF microbiome is commonly cited as a single entity, microbial communities are highly individualized, although some trends are evident [12]. Generally, diversity inversely correlates with lung function as patients with advancing lung disease have communities dominated by core pathogens such as *P. aeruginosa* and *Burkholderia cepacia* complex (*Bcc*) [13, 37, 38]. An individual’s microbiome is relatively constant during clinical stability [12, 13]. However, acute therapies during pulmonary exacerbations with systemic antibiotics are known to induce large, yet transient changes, as the microbiome reverts to baseline following their discontinuation.

While classically accepted pathogens have a clearly documented role in the natural history of CF lung disease, the extent to which microbial communities play a role remains unknown. Indeed, the development and implementation of acute and chronic antibacterial therapies targeting classical pathogens are purported to be one of the major factors responsible for improving clinical outcomes [39]. Three classes of inhaled antimicrobials are commonly used to treat chronic *P. aeruginosa* infection in CF owing to an abundance of supporting clinical data: colistin, tobramycin, and aztreonam [3, 18, 40]. Whereas these agents are typically considered to have a spectrum of activity limited to aerobic Gram-negative organisms based on levels achievable parenterally, aerosolization achieves extremely high concentrations of the drugs specifically to the airways. Indeed, AZLI achieves aztreonam concentrations in sputum of 400–550 μg/ml [7]. Accordingly, we postulated that inhaled antibiotics like their systemic counterparts may induce changes in the microbial communities of the lower airways in individuals with CF and that these communities may influence treatment response. Moreover, it may be possible to use signatures within the microbiome of individuals with CF to predict treatment response to particular CF therapeutics.

In this retrospective study, a cohort of 24 patients with at least two sputum samples collected pre- and post-AZLI treatment initiation were used to analyze changes in the CF microbiota following AZLI therapy. As parenteral aztreonam is not a Health Canada-licensed medication, its use is uncommon in our cohort (~7% of all exacerbations) making the assessment of the impact of AZLI on the microbiome minimally confounded by prior exposure to parenteral aztreonam [21]. Our study demonstrates that although there are no shifts from a gross community perspective, subtle shifts are observed at the genus and OTU level. Moreover, these shifts are not limited to aerobic Gram-negative bacteria suggesting that AZLI may impact anaerobes and Gram-positives as well. Of particular interest, we observed that non-responders consistently have a higher abundance of the obligate anaerobe *Fusobacterium* pre- and post- the initiation of AZLI suggesting that this organism may be associated with lack of response to AZLI. Although *Fusobacterium* has been previously found in the CF microbiota, it has not been associated with outcomes. *Fusobacterium* species have been linked to other human diseases such as periodontal diseases [41] suggesting this organism may have a pathogenic potential and may create a more virulent lower airway community which may result in patients being less responsive to certain therapies. Similarly, in a study conducted by Bradshaw et al. 1998, they found that interspecies interactions between *Fusobacterium nucleatum* and other species enhanced the survival of other obligate anaerobes such as *Prevotella nigrescens* [42]. *Staphylococcus aureus* is now the most prevalent CF pathogen reported in international registries and commonly co-infects with *P. aeruginosa*. Notably, *S. aureus* is highly resistant to the effects of monobactam antibiotics and hence may explain why patients with a higher abundance of *Staphylococcus* are less responsive to AZLI.

Interestingly, we also found in the pre-sample assessment that non-responders had a significantly higher abundance in two OTUs belonging to the *Prevotella* (OTU 41 and 73); however, when we assessed the post-samples, we found that responders now had a significantly higher abundance of *Prevotella* (although belonging to a different OTU 34). Noteworthy, Sherrard et al. 2015 observed that many strains of *Prevotella* derived from CF airways produce extended-spectrum-β-lactamases (ESBLs) [15]. Accordingly, it may be possible that non-responders have a higher abundance of the *Prevotella* isolates that produce ESBLs, which would be effective at degrading antibiotics such as AZLI. Moreover, their work suggests that *Prevotella* isolates may protect *P. aeruginosa* from the effects of antimicrobial agents thereby suggesting that the constituents in the CF microbiota may alter treatment responsiveness.

Similar to the present study, Bernarde et al. [43] found that treatment with Ivacafator a CFTR potentiator (with low level antimicrobial activity) was not associated with significant changes in CF microbiome in a cohort of three individuals but rather subtle changes at the OTU level were observed. Specifically, they found that a *Prevotella* OTU correlated positively with FEV_1_ further supporting the notion that certain constituents in the CF microbiota may be associated with response to different therapies.

Whether other established, guideline recommended CF therapies induce changes in the microbiota is something of considerable interest. Indeed, other inhaled antibiotics such as tobramycin and colistin may also impact the microbiome. Azithromycin has been shown to considerably reduce exacerbation frequency, improve weight gain, and reduce lung function decline in those with chronic *P. aeruginosa* infection [44]. It also has broad activity against microbiome constituents perhaps explaining its importance in CF treatment [45]. Even DNase has been shown to release antimicrobial peptides/defensins bound by sputum DNA and induce changes in cultured organisms recovered following treatment initiation [46].

Despite only 18% of samples being taken from patients during concurrent systemic therapies, marked differences in the microbiota were found when compared to the samples from patients taken off systemic therapies. Both alpha and beta diversity measures were significantly different between samples “on” vs “off” systemic antibiotics, demonstrating community structure of samples changes in response to systemic therapies. Differences were also observed at the genus and OTU levels. These results further support previous findings suggesting that systemic therapies induce significant, albeit transient shifts in the microbiota [12, 13, 17, 22].

Our work supports the notion that the microbiota of CF patients may be associated with gender differences [47]. This was evident in both measured differences in alpha diversity and differences in specific OTUs and genera. Samples taken from males generally had a more diverse microbiome as demonstrated by SDI and lower *P. aeruginosa* abundance. Indeed, these findings are supported by previous works showing females have earlier and higher rates of infection with *P. aeruginosa* [32, 48, 49]. Although, differences were observed in beta diversity using PERMANOVA, when permutations were constrained by patient, these differences were no longer significant suggesting that microbiome data should be analyzed very carefully to avoid misinterpretations due to confounding variables. Larger, multi-centre cohort studies analyses are needed to validate such findings.

Our study has a number of notable limitations. Firstly, we acknowledge that association does not indicate causation. Additional in vivo studies will be needed to support our findings and further understand these complex interactions. Samples were collected as part of an ongoing biorepository based on when patients were in clinic as opposed to a prospectively enrolled study, which would allow for scheduled collection of samples. Future studies looking at the effects of inhaled antibiotic in a cyclical fashion where they are evaluated at pre-determined intervals would be more powerful than our “binning” cycled therapy as on vs off. Moreover, samples collected during the off AZLI cycle may have been on different inhaled antibiotics during chronic sequential treatments therefore potentially confounding our findings. This study is limited by its inclusion of patients with particularly advanced disease—a necessity given AZLI’s role of salvage in Canada [35]. These individuals have significantly less diversity in their microbiome [37, 50], and as such, induced changes would be less apparent than in those with milder lung disease. However, clinical studies have shown AZLI to be particularly effective in individuals with moderate to severe disease, whereas fewer improvements were noted in those with milder disease [5, 6, 9, 18].

Although we have conducted a longitudinal study, we do have limited power to examine all the associations and we acknowledge this for all our interpretations. We did not conduct multivariable analyses accounting for confounders due to sample size limitations but attempted to conduct some stratified analyses when possible. Our primary analysis utilized multiple samples per patient without accounting for the intra-person correlation effects which may have impacted the results in either direction. However, we did incorporate sensitivity analyses including a mixed effects model to account for the repeated measures in the pre- and post-periods for diversity and relative abundance and restricted observation analysis to address the potential impact of multiple measures. The sensitivity analyses were largely consistent with the primary analysis which provides more confidence to study results although we suggest larger prospective studies.

As this is a retrospective study based on clinical encounter data, we are limited as to how much information can be obtained from patient charts. In addition, patients had different start dates with respect to the initiation of AZLI, and the on AZLI samples may have been collected at variable time points making it challenging to detect differences. Another limitation is the use of sputum as our sole sample type given the potential for oral flora to contaminate sputum. Importantly, however, sputum remains an easily accessible non-invasive sample from the lower airways and, regardless of contamination, can be harnessed as a relevant biomarker. Other sample types such as a bronchoalveolar lavage may help reduce biases and false positives and be more representative of the spatial heterogeneity that exists in the CF lung microbiome but poses risk.

Our data fails to assess the resistome of the lower airways community, something that currently is not well understood. For us to better explain the changes that occur in the microbiome with antibacterial exposures, a detailed understanding of the antibiogram of constituent microbiota from the CF lung is required. Indeed, preliminary data suggest that “normal flora” derived from individuals with CF are markedly more resilient to the effects of antibiotics relative to those derived from normal populations, infrequently exposed to antibiotics [16].

The present cohort and CF patients in general experience a tremendous treatment burden with polypharmacy, and multiple other antibiotics, respiratory therapies, and nutritional and gastrointestinal supplements, that may further confound our findings. Many studies have reported adherence is poor in CF and inversely associates with treatment burden [51, 52]. Given samples were collected as part of an ongoing clinical observational study, we are not able to determine medication compliance beyond what patients self-report to care providers and is documented in the clinical record. Indeed, adherence rates may be different between groups (i.e., responders versus non-responders) and therefore potentially biasing our findings.

Future studies assessing investigational CF agents would be well advised to prospectively incorporate sputum, serum, and urine biobanking of specimens that would allow for a retrospective analysis of factors associating with treatment outcomes including the microbiome, metagenome, metabolome, infammasone, and resistome as technological advancements continue.

## Conclusions

Results from our study suggest that the CF microbiome is relatively resilient to AZLI perturbations. Although no significant changes were observed in overall diversity measures after initiation of AZLI, differences were observed at the genus and OTU level suggesting subtle shifts in certain constituents of the microbiome may occur because of AZLI therapy. Lack of response to AZLI was observed in patients whose sputum had a higher abundance of OTUs belonging to *Fusobacterium*, *Staphylococcus*, *Prevotella*, and *Bacteroides*. Further studies assessing treatment response in the context of pre-therapy microbiome signatures may enable personalization of therapy. Such a strategy would enable improved patient outcomes and avoid the negative economic and health outcomes associated with prescribing a less effective therapy*.*

## Additional file

Additional file 1: Figure S1. Schematic representation of a theoretical patient’s biobanked samples of frozen whole sputum relative to initiation of AZLI. Days from initiation of AZLI are listed on the x-axis. Samples could have been collected at any time point during the 1 year prior to and subsequent to AZLI initiation and samples may have been collected on/off therapy as treatments are administered in 28 day cycles or during cycling of other inhaled antibiotics. Samples may have also been collected during the use of systemic antibiotics. Prior Inh-Abx refers to prior chronic suppressive antibacterial treatment. (TIF 856 kb)

## Abbreviations

AZLI: Aztreonam Lysine for Inhalation
BC: Bray Curtis
Bcc: *Burkholderia cepacia* complex
BMI: Body mass index
CACFC: Calgary Adult CF Clinic
CF: Cystic fibrosis
CFU: Colony-forming unit
DIOS: Distal intestinal obstruction syndrome
DNA: Deoxyribonucleic acid
FEV1: Forced expiratory volume in one second
FVC: Forced vital capacity
IQR: Interquartile range
NSAID: Non-steroidal antiinflammatory drug
OTU: Operational taxonomic unit
PERMANOVA: Permutational multivariate analysis of variance
rRNA: Ribosomal RNA
SD: Standard deviation
SDI: Shannon diversity index
TIP: Tobramycin inhalation powder
TIS: Tobramycin inhalation solution
