# Supplementary figures and images for: The effects of inhaled aztreonam on the cystic fibrosis lung microbiome
Source: Microbiome. 2017 May 5;5:51. doi: 10.1186/s40168-017-0265-7 (PMC5420135; doi:10.1186/s40168-017-0265-7)

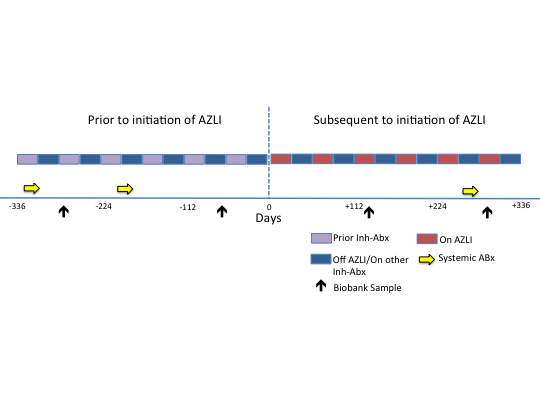

Supplement: Additional file 1: Figure S1. — Schematic representation of a theoretical patient’s biobanked samples of frozen whole sputum relative to initiation of AZLI. Days from initiation of AZLI are listed on the x-axis. Samples could have been collected at any time point during the 1 year prior to and subsequent to AZLI initiation and samples may have been collected on/off therapy as treatments are administered in 28 day cycles or during cycling of other inhaled antibiotics. Samples may have also been collected during the use of systemic antibiotics. Prior Inh-Abx refers to prior chronic suppressive antibacterial treatment. (TIF 856 kb) [file 40168_2017_265_MOESM1_ESM.tif]
